# Effects of Phytochemicals on Blood Pressure and Neuroprotection Mediated Via Brain Renin-Angiotensin System

**DOI:** 10.3390/nu11112761

**Published:** 2019-11-14

**Authors:** Hye Lin Kim, Woo Kyoung Kim, Ae Wha Ha

**Affiliations:** 1 Department of Food Science and Nutrition, Dankook University, 119, Dandae-ro, Dongnam-gu, Cheonan-si, Chungnam 31116, Korea; gpfls7085@naver.com (H.L.K.); wkkim@dankook.ac.kr (W.K.K.); 2 Natural Nutraceuticals Industrialization Research Center & Department of Food Science and Nutrition, DanKook University, 119, Dandae-ro, Dongnam-gu, Cheonan-si, Chungnam 31116, Korea

**Keywords:** curcumin, saponin, quercetin, blood pressure, renin angiotensin system, acetylcholine

## Abstract

Background: The renin-angiotensin system (RAS) in the brain plays a crucial role in maintaining blood pressure as well as neuroprotection. This study compared the effects of curcumin, quercetin, and saponin on blood pressure, the brain RAS, and cholinergic system using perindopril, an angiotensin converting enzyme inhibitor (ACEI), as a positive control. Methods: Five-week-old male mice were stabilized and randomly assigned into a control group (*n* = 8), three phytochemical-treated groups (curcumin (*n* = 8), quercetin (*n* = 8), and saponin (*n* = 8)), and a positive control group (*n* = 8). The groups treated with the phytochemical were orally administered daily at a dose of 50 mg/kg body weight of phytochemicals. During the experiments, the weight and dietary intakes were measured regularly. After experiments, the brain tissue was homogenized and centrifuged for an additional assay. The concentrations of ACE, angiotensin II (AngII), and aldosterone levels were measured, and the mRNA expressions of renin and ACE were measured. As biomarkers of neuroprotection, the concentrations of acetylcholine (ACh) as well as the concentration and activity of acetylcholine esterase (AChE) were measured. Results: After 4 weeks of treatment, the perindopril group showed the lowest blood pressure. Among the groups treated with the phytochemicals, treatment with curcumin and saponin significantly reduced blood pressure, although such effect was not as high as that of perindopril. Among phytochemicals, curcumin treatment significantly inhibited the concentration and activity of ACE, concentration of AngII, and mRNA expression of ACE. All phytochemical treatments significantly increased the concentration of ACh. The levels of AChE activity in groups exposed to curcumin or saponin (not quercetin) were significantly inhibited, Conclusion: Curcumin administration in rats reduced blood pressure by blocking the brain RAS components and protected the cholinergic system in brain by inhibiting the activity of AChE.

## 1. Introduction

The renin-angiotensin system (RAS) plays an important role in the regulation of blood pressure [1]. When blood pressure is low, renin is secreted from cells in the kidney. Renin converts angiotensinogen in blood to angiotensin I (AngI). Angiotensin converting enzyme (ACE) converts AngI to angiotensin II (AngII), which is a strong vasoconstrictor. Increased production of AngII results in constriction of arterial blood vessels and induces the release of aldosterone from adrenaline, thereby increasing the blood pressure [2]. 

RAS is present not only in the periphery, but also in various tissues such as kidneys, adipose tissue, and the brain [1]. In brain, the blood-brain barrier (BBB) restricts peripheral RAS components from accessing the majority of brain regions, making the local synthesis of brain RAS essential. Substantial amounts of ACE, AngI, and AngII have been detected in brain tissue [3,4]. 

Both peripheral and brain RAS production contribute significantly to cardiovascular homeostasis. However, chronic activation of RAS components leads to oxidative stress, endothelial dysfunction, and inflammation, leading to a number of diseases, including hypertension. Clinical studies have shown that treatment with RAS component inhibitors (especially ACE inhibitor) in patients with hypertension improves cognitive function, in addition to blood pressure-lowering effects [5,6,7].

Cholinergic neuronal signaling in the brain modulates various aspects of cognition, learning, and memory [8]. Acetylcholinesterase (AChE or acetylhydrolase) in the brain degrades acetylcholine (ACh) into choline and acetic acid. Decrease in acetylcholine receptor and increase in acetylcholinesterase activity deplete the neurotransmitter ACh in Alzheimer’s disease (AD) [9]. Thus, substances that inhibit both ACE and AChE can be used to protect cognitive function in patients with hypertension-related diseases.

ACE inhibitors such as perindopril, enalapril, and ramipril are widely used to treat hypertension [7]. Perindopril is a more lipophilic compound and easily diffuses through the BBB compared with other ACE inhibitors [5]. Studies showed that the active metabolites of perindopril were more highly distributed in the rat brains compared with other ACE inhibitors. In human studies, perindopril reduced the risk of dementia and cognitive dysfunction in patients with hypertension-related diseases [5,6]. In several animal studies, perindopril showed ACE inhibition in the brain, which in turn attenuated Ang II formation (lower blood pressure) as well as restored the decreased AChE activity [10,11,12,13]. 

However long term use of hypertension medications could cause some side effects such as altered taste, leukocytopenia, angioedema, liver dysfunction, and dry cough [14]. Therefore, a study to identify safe and active substances with blood pressure-lowering and brain-protective function is essential. Among the various phytochemicals found in plants, some phenolic compounds can cross the BBB and appear to exhibit neuroprotective and clinical potential in the central nervous system [15,16,17,18]. Two main groups of neuroprotective plant chemicals include diarylheptanoids (curcumin) and flavonoids (quercetin). Diarylheptanoids and flavonoids exhibit neuroprotective effects and protect neurons from damage especially via free-radical scavenging activity [15,16]. The active ingredient in ginseng is saponin (especially Rg1 or Rg2). Saponin was detected in homogenized brain tissue, and was dispersed throughout vascular endothelial cells, astrocytes, and neurons suggesting that saponin can permeate through BBB after oral administration [18]. 

To date, studies reported the role of phytochemicals and hypertension or cognitive protection [15,16,17,18]. However, no studies investigated the role of phytochmicals in blood pressure and neuroprotection by regulating brain RAS. Therefore, in this study, the effects of oral administration of curcumin, quercetin, and saponin on the brain RAS and cholinergic system were compared with perindopril as a positive control (ACE inhibitor).

## 2. Materials and Methods

### 2.1. Experimental Animals 

Five-week-old ICR mice (male, *n* = 40) were purchased from Asan Life Research Center (Seoul, Korea) and were acclimated to the environment for 1 week. Each mouse was placed in a standard cage, with water and AIN-93G standard diet provided ad libitum. During the experimental period, mice were raised at a temperature of 23 ± 3 °C, relative humidity 50% ± 20%, ventilation frequency of 10–20 /h, illumination time of 12 h and optimal illumination intensity. The animals received the approval of Dankook University Animal Experiment Ethics Committee. The experiment was conducted according to the Guidelines of the Animal Experiment Ethics Committee of Dankook University (approval number: 16-023). 

### 2.2. Experimental Design and Treatment

After stabilization, the ICR mice were randomly assigned to a control group (*n* = 8), three groups treated with phytochemicals including curcumin (*n* = 8), quercetin (*n* = 8), and saponin (*n* = 8), respectively. A positive control group was also established (*n* = 8). Curcumin, quercetin, and saponin were orally administered daily at a dose of 50 mg/kg body weight regularly. In the positive control group, perindopril was orally administered daily at a dose of 1 mg/kg body weight, under similar conditions as the phytochemical-treated groups. The test materials, quercetin (95%), saponin (8–25%), curcumin (Curcuma longa L., 65%) and perindopril were purchased from Sigma (Sigma Aldrich, St. Louis, MO, USA). Curcumin, quercetin, saponin and perindopril were dissolved in 0.1% Tween 80 (Sigma-Aldrich Co., St. Louis, MO, USA) according to the daily dose immediately prior to administration. The control group was treated with the same amount of physiological saline. 

### 2.3. Weight Gain, Feed Intake, and Assay Sampling Procedure

During the experimental period, the body weight was measured once every two days, and the dietary weight was measured once every three days at a constant time frame. Weight gain was calculated by subtracting the initial weight from the final body weight and dividing it by the total breeding period. Food efficiency ratio (FER) was calculated by dividing the total weight gain by the total food intake for 4 weeks. 

Before the end of the experiment, animals were fasted for 12 h, anesthetized, and dissected. The collected blood from the heart was separated by centrifugation at 3000 rpm for 15 min (Gyrozen, Daajeon, Korea). After collecting blood, brain, liver, kidney, spleen, thymus, epididymal fat, visceral fat, and kidney fat were extracted, washed with 0.9% NaCl solution, and weighed after removing the water. A cold sodium phosphate buffer (pH 7.2–7.4) was added to the excised brain tissue, which was then homogenized and centrifuged for 5 min at 5000 g and 4 °C. 

### 2.4. Blood Pressure Measurement

Blood pressure was measured at 0, 2, and 4 weeks of the experiment. In order to reduce the error range of blood pressure measurement, a laboratory animal was calibrated in a holder of a BP-2000 blood pressure analysis system (rat platform 2-channel, Visitech Systems, Apex, NC, USA), and acclimated in a 37 °C heating chamber for 30 min. Blood pressure was repeatedly measured three times. After the tail artery was fully extended, occlusion cuffs were placed over the upper tail, which was attached to the pneumatic pulse sensor connected to the physiograph. The pulses generated while inflating the cuff and depressurizing were recorded in the physiograph.

### 2.5. Renin Angiotensin System (RAS) Measurement

#### 2.5.1 Angiotensin converting enzyme (ACE), angiotensin II, and aldosterone

The concentration of ACE was measured by using ACE ELISA KIT (MyBioSource, San Diego, CA, USA). ACE activity was measured using the ACE diagnostic kit (Biovision, Mountain View, CA, USA) and fluorescence measurement in homogenates of brain tissues. To measure the concentration of angiotensin II and aldosterone, the brain tissue homogenate was pre-treated with the ELISA kit (MyBioSource, San Diego, CA, USA) and the absorbance was measured at 450 nm using a microplate reader (TECAN, Port Melbourne, Austria). 

#### 2.5.2 Transcription of renin and ACE 

Some of the brain tissues were homogenized with Tris reagent, and mRNAs were extracted with chloroform, isopropanol, and 75% ethanol. SuperScript kit (Invitrogen, Carlsbad, CA, USA) was used to reverse-transcribe the extracted mRNA to cDNA. Oligo DT, reaction mixture (10 × RT buffer, 0.1 M DTT, 10 mM NTP), superscript II reverse transcriptase, and RNase H were sequentially added, incubated, and synthesized as cDNA. Prepared cDNA samples, SYBP Green Master Mix (Applied Biosystems, Foster City, CA, USA), and nuclease-free water and the following forward/reverse primers were mixed: GAPDH (sense: ATCAAATGGGGTGATGCTGGTGCTG/antisense: CAGGTTTCTCCAGGCGGCATGTCAG); Renin (sense: AGGCAGTGACCCTCAACACCAG/antisense: CCAGTATGCAGGTCGTTCCT); ACE (sense: GCCTCCCCAACAAGACTGCCA/antisense: CCACATGTCTCCCCAGCAGATG). The reaction was conducted using Applied Biosystems StepOne (Applied Biosystems, Foster City, CA, USA) at 95 °C. Using software v2.1, the mixture was reacted for 10 min at 95 °C, 15 min at 95 °C, 1 min at 60 °C, 15 min at 95 °C, 1 min at 60 °C, and 15 min at 95 °C for 40 cycles. The results were calculated using a built-in program of Applied Biosystems StepOne (Apllied Biosystems, Foster City, CA, USA) software v2.1 via △△CT method. GAPDH (Glyceraldehyde-3-phosphate dehydrogenase) was used as a loading control under the same experimental conditions. The experiment was repeated three times. 

### 2.6. Cholinergic System Measurement

The concentration of acetylcholine was determined using Acetylcholine ELISA kit (MybioSource, San Diego, CA, USA). The standard solution and brain tissue homogenates were dispensed into a prepared 96-well plate, and 10 μL of balance solution and 50 μL of conjugate were dispensed into each well, followed by incubation at 37 °C for 1 h. The solution was washed and 100 μL of substrate solution was added to induce color development. The absorbance was measured using a microplate reader (TECAN, Port Melbourne, VIC, Austria) at 450 nm. 

The concentration of acetylcholine esterase was determined using an acetylcholine esterase ELISA kit (Cusabio, Balitmore, MD, USA). After all the treatments specified for the assay, TMB substrate solution was added to develop the color and the absorbance was measured at 450 nm using a microplate reader (TECAN, Port Melbourne, VIC, Austria). The activity of acetylcholine esterase in brain tissue homogenates was determined using the acetylcholineesterase assay kit (BioAssay System, Hayward, CA, USA). The enzyme activity was measured at 412 nm at intervals of 2 and 10 min. 

### 2.7. Statistical Analysis

All data derived from this study were analyzed using SPSS Statistics 23.0 (SPSS Inc., Chicago, IL, USA) and expressed as the mean and standard error. One-way analysis of variance (ANOVA) was conducted to determine significant differences between the groups. Duncan’s multiple comparison test was conducted to verify the respective variation in each group compared with the control, and significant group differences (*p* < 0.05) were determined using ANOVA.

## 3. Results

### 3.1. Weight Change, Food Intake, and Food Efficiency Ratio (FER)

The body weight, food intake, and food efficiency ratio (FER) are shown in Table 1. There was no significant difference in the initial body weight among the groups. However, there was a significant difference in final weight between control group and saponin group (*p* < 0.05). Weight gain over the 4 weeks was significantly lower in the perindopril, curcumin, or saponin groups when compared with that of the control group (*p* < 0.05). FER of the saponin group was significantly differenent compared with the control group (*p* < 0.05).

### 3.2. Phytochemical Treatments and Changes in Blood Pressure

Systolic blood pressure was not significantly different among treatment groups at week 0 (Table 2). However, in all treatment groups except quercetin group, systolic blood pressure at week 2 was significantly lower than in the control group (*p* < 0.05). After 4 weeks of treatment, the systolic blood pressure of the perindopril group was the lowest and showed a significant difference from the control group (*p* < 0.05). or the other phytochemical groups (*p* < 0.05). Among the groups exposed to phytochemicals, the curcumin (*p* < 0.05) and saponin group (*p* < 0.05) showed a significant decrease in systolic blood pressure compared with that of the control group. In all phytochemical groups, no significant difference in diastolic blood pressure, compared to control or perindopril, was found.

### 3.3. Effect on the Renin Angiotensin System (RAS) 

#### 3.3.1. Angiotensin Converting Enzyme (ACE) Concentration and ACE Activity in Brain

**Figure 1:**
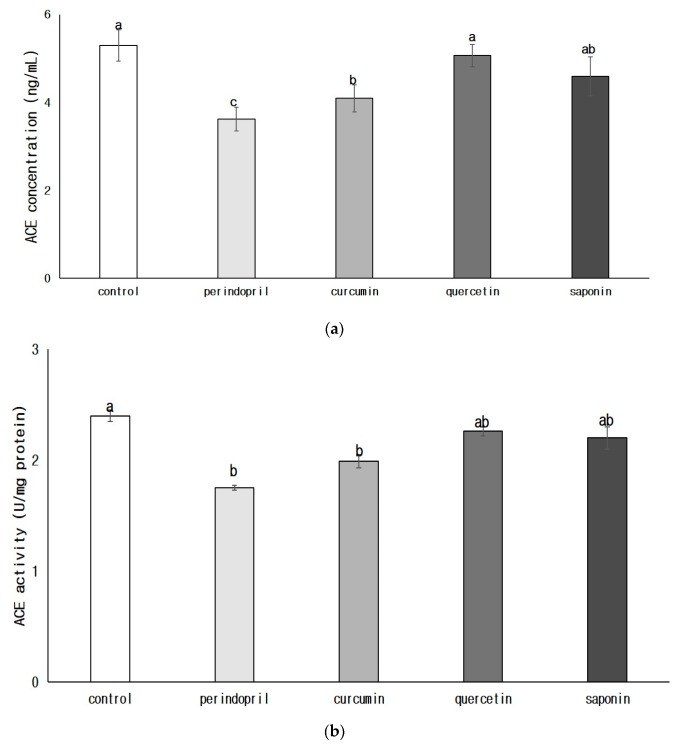
Effects of phytochemicals on angiotensin converting enzyme (ACE) concentration (**a**) and ACE activity (**b**) in brain. Control: 0.9% saline oral administration; Perindopril: oral administration of 1 mg perindopril/kg body weight; Curcumin, Quercetin, Saponin: oral administration of 50 mg of each phytochemical/kg body weight; Different letters indicate significant differences at *α* = 0.05 as determined by Duncan’s multiple range test.

The concentration of ACE in brain was significantly decreased in the perindopril group compared with the control group (*p* < 0.05) or any phytochemical groups (*p* < 0.05). (Figure 1). Both ACE concentration and ACE activity significantly decreased in the curcumin group, compared to the control group (*p* < 0.05). The ACE concentration and ACE activity in quercetin and saponin groups were not significantly different from that of the control group.

#### 3.3.2. Concentrations of Angiotensin II (AngII, a) and Aldosterone (b) in Brain 

**Figure 2:**
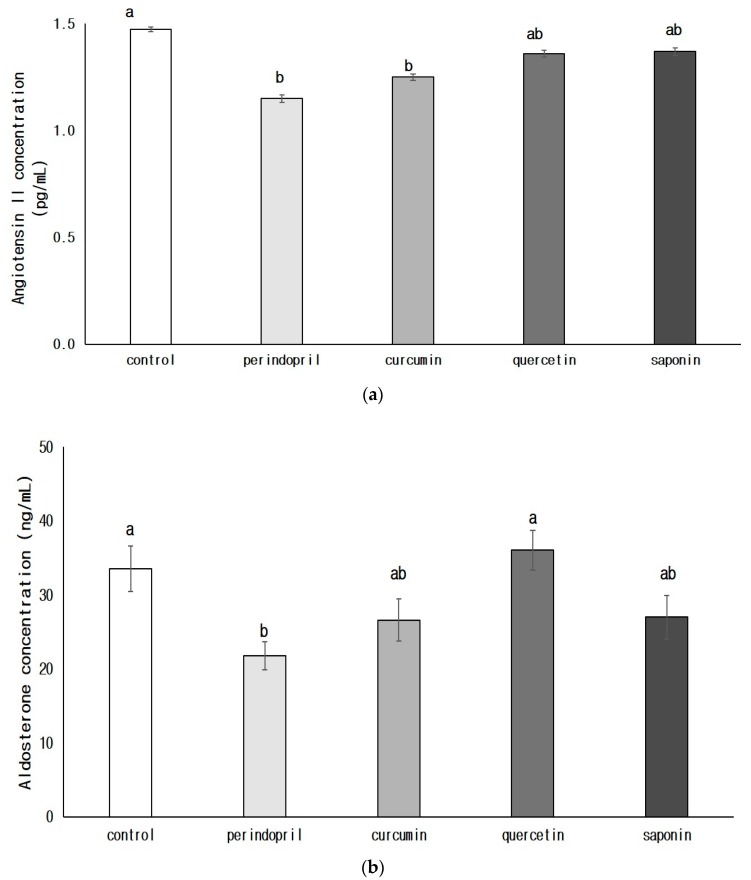
Effects of phytochemicals on the concentrations of angiotensin II (**a**) and aldosterone (**b**) in brain. Control: 0.9% saline oral administration; Perindopril: oral administration of 1 mg perindopril/kg body weight; Curcumin, Quercetin, Saponin: oral administration of 50 mg of each phytochemical/kg body weight; Different letters indicate significant differences at *α* = 0.05 as determined by Duncan’s multiple range test.

The concentration of brain angiotensin II in the perindopril group (*p* < 0.05) or curcumin group (*p* < 0.05) was significantly lower than that of the control group (Figure 2). The amount of aldosterone significantly decreased in the perindopril group compared with that of the control group (*p* < 0.05). Among phytochemical groups, aldosterone concentrations in curcumin or saponin group were lower than in the control group; however, there was no statistical significance.

#### 3.3.3. Transcription of Renin and ACE in the Brain

**Figure 3:**
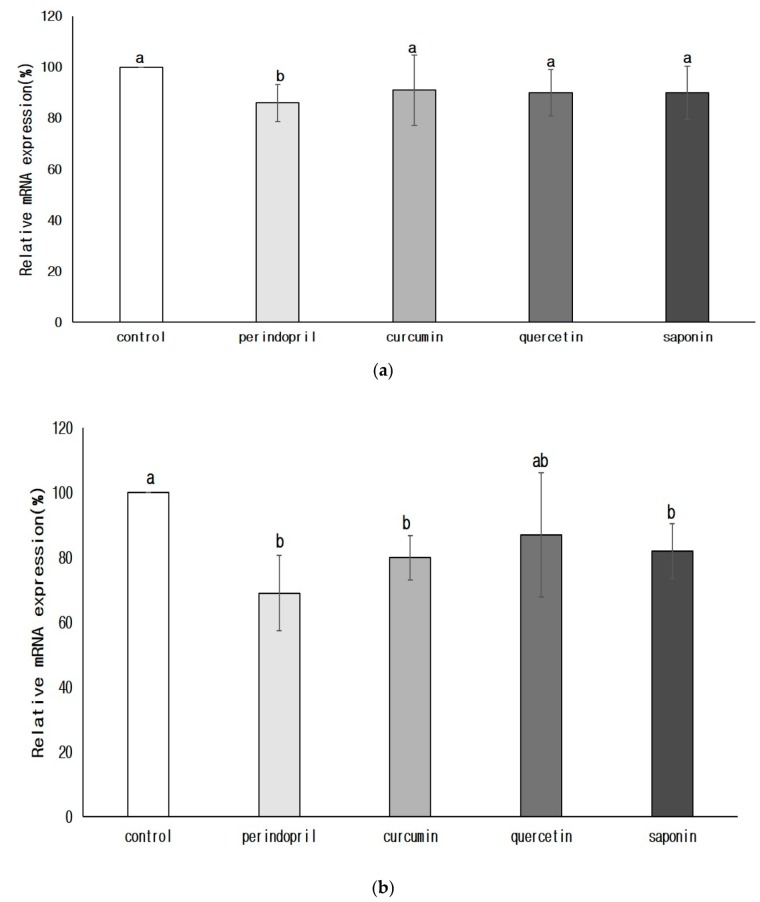
Effects of phytochemicals on the mRNA expression of renin (**a**) and angiotensin converting enzyme (**b**) in brain. Control: 0.9% saline oral administration; Perindopril: oral administration of 1 mg perindopril/kg body weight; Curcumin, Quercetin, and Saponin: oral administration of 50 mg each phytochemical/kg body weight; Different letters indicate significant differences at *α* = 0.05 as determined by Duncan’s multiple range test.

The mRNA expression of renin and ACE was significantly lower in the perindopril group than in the control group (*p* < 0.05) (Figure 3). The degree of renin expression in the groups treated with phytochemical was not significantly different from that of the control group. However, ACE mRNA expression was significantly lower in the curcumin group (*p* < 0.05) or in the saponin group (*p* < 0.05) than in the control group. There was no significant difference in ACE mRNA expression between quercetin and control group or perindopril group. 

### 3.4. Cholinergic Expression in the Nervous System 

**Figure 4:**
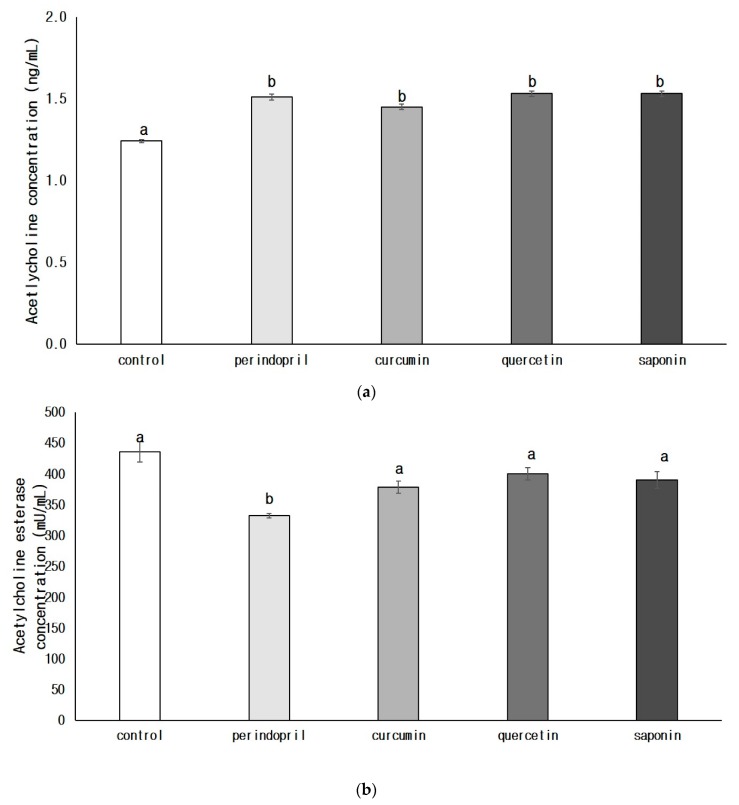

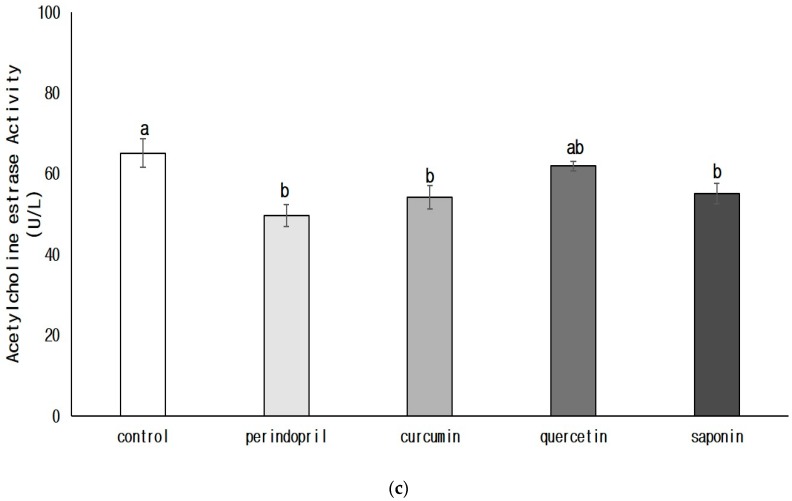
Effects of phytochemicals on the concentrations of acetyl choline (**a**) and acetylcholine esterase (**b**) and acetylcholine esterase activity (**c**) in the brain. Control: 0.9% saline oral administration; Perindopril: oral administration of 1 mg perindopril/kg body weight; Curcumin, Quercetin, and Saponin: oral administration of 50 mg each phytochemical/kg body weight; Different letters indicate significant differences at *α* = 0.05 as determined by Duncan’s multiple range test.

The ACh concentration was significantly increased in the perindopril group (*p* < 0.05) or in all phytochemical groups (*p* < 0.05) when compared with that of the control group (Figure 4a). The concentration of acetylcholine esterase was significantly lower only in the perindopril group than in the control group (*p* < 0.05) (Figure 4b). The levels of AChE activity in groups exposed to perindopril (*p* < 0.05), curcumin (*p* < 0.05), or saponin (*p* < 0.05) were significantly inhibited, when compared with that of the control group (Figure 4c). There was no significant difference of AChE activity between quercetin and control group or perindopril group.

## 4. Discussion

Inhibition of excessive activation of RAS in the brain not only helps in controlling blood pressure but also improves cognitive function [4]. ACE inhibitors, especially perindopril, appear to be effective in reducing hypertension and enhancing cognitive protection by modulating brain RAS [5,6]. To date, no study has investigated the role of phytochemicals in regulating both blood pressure and neuroprotection via brain RAS. Thus, in this study, using perindopril (ACE inhibitor) as a positive control, the effects of various phytochemicals including curcumin, quercetin, and saponin on brain RAS and the cholinergic system, were studied.

After 4 weeks of treatment with either phytochemical or perindopril, the results of this study showed that the administration of perindopril was the most effective. The hypotensive effect of perindopril was due to the inhibition of ACE levels and its mRNA expression of ACE, which further inhibit angII and aldosterone concentrations. In this study, the dosage of perindopril treatment used was 1 mg/kg body weight, similar concentration range, which demonstrated to inhibit more than 50% of hippocampal ACE in previous studies [10,11,12,13,14]. Perindopril is an effective ACE inhibitor in brain because it penetrates the BBB and effectively increases cerebral blood flow [11]. Perindopril treatments in humans [5,6] and animal studies [10,11,12,13,14] showed a significant reduction in the conversion of Ang I to Ang II, ultimately leading to the decreased synthesis of Ang II. Excessive brain RAS activity, especially Ang II, is responsible for the development and maintenance of hypertension [19].

Treatment with curcumin was significantly more effective than the control in reducing the blood pressure and inhibiting ACE activation and ACE mRNA expression (*p* < 0.05) but less effective than perindopril treatment. Curcumin is a physiologically active substance found in turmeric (Curcuma longa L.). Turmeric is a perennial herbaceous plant belonging to the ginger family, and is primarily cultivated in India, Taiwan, Indonesia, and Japan [20]. Past studies investigating the cardioprotective effect of curcumin have focused on its antioxidant activity; Curcumin suppresses heart damage caused by antioxidant activity and prevents deterioration of systolic function in hypertensive heart disease [21,22,23,24]. However, our study suggests that curcumin has an antihypertensive effect due to inhibition of ACE activation in the brain. Curcumin-induced brain ACE inhibition demonstrated in this study decreased the synthesis of brain Ang II and further reduced blood pressure. Although limited studies exist, our findings are consistent with the below studies [25,26,27]. Curcumin reduced AT1R expression, thereby reducing Angiotensin II, AT1R-mediated vasoconstriction, and subsequently suppressed hypertension in Ang II-induced hypertension models [25], and in vascular smooth muscle cells [26]. When rats were exposed to both curcumin (75 mg/kg weight) and thioacetamide (TAA)-induced hepatotoxicity for 8 weeks, the ACE gene expression in brain was significantly reduced when compared with TAA-induced control group [27]. Overall, our results together with those reported in earlier studies [25,26,27] indicate that curcumin prevents the development of hypertension by regulating brain RAS.

The second objective of this study was to determine whether phytochemicals, when compared with perindopril, have additional benefits on the cholinergic system in brain, besides blood pressure control. In brain tissues, choline and acetyl CoA are converted into acetylcholine by acetyltransferase, and ACh is converts into acetate and choline by AChE. The increased AChE activity leads to memory loss due to decreased ACh levels [9]. In this study, the administration of perindopril significantly increased ACh concentration in the brain, and decreased AChE concentration and AChE activity. These results are consistent with previous studies showing that perindopril was the most centrally active AChE inhibitor and suppressed brain AChE activity; it increased ACh concentration in an animal model of vascular dementia [14,15,16]. Clinical trials have also suggested that perindopril slowed down the cognitive decline in hypertensive patients with AD or reduced the risk of AD in elderly patients with hypertension [5,6].

Phytochemicals, especially curcumin or saponin, also significantly reduced AChE activity compared with the control, although not as effectively as perindopril. Curcumin has been shown to protect against neurodegenerative diseases via antioxidant effects in rats with AD induced with or without stress [28,29]. Compared with previous studies, this study showed that curcumin had neuroprotective effects possibly via inhibition of brain ACh and AChE activity. When curcumin was administered to mercury-intoxicated pregnant rats, the levels of dopamine, serotonin, and AChE in the brain were lower in the newborn rats compared with the control group [30]. A direct link between brain RAS and the cholinergic system has also been established [4]. As high AngII in brain inhibits acetylcholine release, ACE inhibitors may enhance cognition via release of ACh [3]. Hence, the combination of curcumin treatment with ACE inhibitors, especially perindopril for the inhibition of ACE and AChE activity in the brain is a better intervention in hypertensive patients with the risk of vascular dementia.

Saponin is a major bioactive substance of ginseng, a perennial plant belonging to the genus *Panax* of Araliaceae [18]. Saponin treatment in spontaneously hypertensive rats (SHR) reduced systemic blood pressure and blocked the circulating and tissue RAS [31]. The major components of saponin, Rg1, and Rg2, have been reported to improve learning and memory in experimental animals with electrically impaired memory and in animals with drug-induced memory damage. [32,33]. A 4-week oral administration of saponin in this study also lowered blood pressure and decreased both ACE activity and AChE activity in brain. However, dietary intake and dietary efficiency ratio were significantly lower in the saponin group than in the other phytochemical groups or the control group. Thus, there is a possibility that the antihypertensive effect following saponin administration may be related to the decreased dietary intake, and further studies are needed to investigate this finding. 

The study limitations are as follows. Since this study investigated to compare the effect of phytochemicals in lowering blood pressure and neuroprotection mediated via brain RAS and cholinergic system, a single dose of various phytochemicals (50 mg/kg) was orally administered in rats. A further study is needed to investigate the protective effects of phytochemicals at various concentrations on a hypertensive target organ in SHR rats. Additionally, we did not measure the concentration of each phytochemical in the brain after 4 weeks of treatment. However, this study used perindopril, an antihypertensive drug (a known ACE inhibitor), as a positive control. Under the same experimental condition, perindopril demonstrated a potent antihypertensive effect. Therefore, we believe that the protective effect of curcumin under the same conditions was not an error of the experimental method. Despite the study limitations, to our knowledge, this is the first investigation to demonstrate the protective effect of curcumin against both blood pressure and cholinergic system via modulation of brain RAS. 

## 5. Conclusions

Curcumin lowered blood pressure via inhibition of ACE and AngII level and protected the brain cholinergic system by inhibiting AChE activity. Thus, we speculate that the inhibition of brain RAS by curcumin not only affects blood pressure but also cognitive function. Therefore, curcumin is expected to be a beneficial phytochemical for the management of hypertensive patients at risk of cognitive decline.

## Figures and Tables

**Table 1:** Body weight gain, food intake, and food efficiency ratios.

Group ^1)^	Initial Weight (g)	Final Weight (g)	Weight Gain (g/4 Weeks)	Food Intake (g/4 Weeks)	FER ^2)^
Control	26.4 ± 0.9 ^3)NS4)^	34.6 ± 1.2 ^a5)^	8.2 ± 1.9 ^a^	105.8 ± 4.4 ^a^	0.08 ± 0.02 ^a^
Perindopril	26.8 ± 1.1	33.8 ± 2.1 ^a^	7.1 ± 2.1 ^b^	100.8 ± 8.6 ^a^	0.06 ± 0.02 ^ab^
Curcumin	26.7 ± 1.1	32.5 ± 1.9 ^ab^	6.0 ± 1.6 ^bc^	97.7 ± 7.4 ^ab^	0.06 ± 0.02 ^ab^
Quercetin	26.9 ± 0.9	34.2 ± 2.1 ^a^	7.3 ± 1.8 ^ab^	101.2 ± 4.8 ^a^	0.06 ± 0.02 ^ab^
Saponin	26.7 ± 1.0	31.5 ± 2.4 ^b^	5.6 ± 2.0 ^c^	96.0 ± 8.4 ^b^	0.05 ± 0.02 ^b^

^1)^ Control: 0.9% saline; perindopril: perindopril (1 mg/kg); curcumin, quercetin, and saponin groups: (50 mg/kg); ^2)^ FER (food efficiency ratio) = Body weight gain for 4 weeks/Food intake for 4 weeks; ^3)^ mean ± standard error (SE); ^4)^ NS: not significant by ANOVA test. ^5)^ Different letters in a row indicate significant differences at α = 0.05 as determined by Duncan’s multiple range test.

**Table 2:** Effects of phytochemicals on systolic and diastolic blood pressure.

Group ^1)^	Systolic Blood Pressure (mmHg)			Diastolic Blood Pressure (mmHg)		
	0 Weeks	2 Weeks	4 Weeks	0 Weeks	2 Weeks	4 Weeks
Control	98.8 ± 19.3 ^2)NS3)^	125.0 ± 9.1 ^a4)^	113.3 ± 10.4 ^a^	53.2 ± 11.4 ^NS^	63.6 ± 26.8 ^NS^	57.7 ± 13.7 ^NS^
Perindopril	111.1 ± 20.4	104.2 ± 5.4 ^b^	78.1 ± 17.9 ^b^	65.4 ± 22.8	46.1 ± 13.0	61.1 ± 19.9
Curcumin	103.3 ± 11.6	108.8 ± 15.2 ^b^	95.4 ± 21.3 ^c^	44.5 ± 16.9	54.4 ± 16.9	48.3 ± 25.8
Quercetin	106.6 ± 9.0	118.5 ± 14.5 ^b^	102.6 ± 1.3 ^ac^	50.0 ± 22.6	57.6 ± 22.0	58.9 ± 24.7
Saponin	99.1 ± 14.5	109.6 ± 10.4 ^b^	97.6 ± 7.3 ^c^	62.9 ± 20.0	58.3 ± 21.3	48.3 ± 12.2

^1)^ Control: 0.9% saline; perindopril: perindopril (1 mg/kg); curcumin, quercetin, and saponin (50 mg/kg); ^2)^ mean ± standard error (SE); ^3)^ NS: not significant by ANOVA test. ^4)^ Different letters in a row indicate significant differences at α = 0.05 as determined by Duncan’s multiple range test.
